# The role of reed management and habitat quality on brood parasitism and chick survival of the brood parasitic Common Cuckoo

**DOI:** 10.1002/ece3.9705

**Published:** 2023-01-03

**Authors:** Thomas Oliver Mérő, Antun Žuljević, Szabolcs Lengyel

**Affiliations:** ^1^ Institute of Aquatic Ecology Centre for Ecological Research Debrecen Hungary; ^2^ Nature Protection and Study Society – NATURA Sombor Serbia

**Keywords:** agricultural landscape, bio‐indicator, habitat management, nest success, reedbed

## Abstract

Despite efforts on ecosystem restoration and management, biodiversity loss remains one of the major environmental concerns of our time. Beyond the focus on threatened species, animals that indicate regional biodiversity hotspots and population trends, such as brood parasites, should also be targeted by conservation actions. We studied how reed habitat quality and management influence brood parasitism rate and offspring survival in Common Cuckoos *Cuculus canorus* parasitizing nests of Great Reed Warblers *Acrocephalus arundinaceus* in six reed habitats in an intensive agricultural landscape. Data collected from 45 sites over 13 years showed that the brood parasitism rate was highest on large canals and was positively influenced by the availability of potential perches (Cuckoo vantage points) and the height where host nests were built. Cuckoo chick survival decreased with water depth and was not affected by other factors. Our results suggest that the habitat‐dependent detectability of host nests was central in brood parasitism rate and that water level was central in Cuckoo chick survival. Our study shows that a maintenance of intermediate water levels is the most optimal for maintaining Cuckoo populations in intensive agricultural landscapes. Because brood parasites are excellent bioindicators as their presence predicts regional hotspots of taxonomic and functional diversity as well as population trends in bird communities, knowledge on their habitat requirements is relevant in management targeting diverse bird communities.

## Graphical abstract

We studied how reed habitat quality and management influence brood parasitism rate and offspring survival in Common Cuckoos parasitizing nests of Great Reed Warblers in six reed habitats in an intensive agricultural landscape. Brood parasitism rate was highest on large canals and was positively influenced by the availability of potential perches, while the Cuckoo chick survival decreased with water depth and was not affected by other factors. Our results suggest that the habitat‐dependent detectability of host nests was central in brood parasitism rate and that water level was central in Cuckoo chick survival.

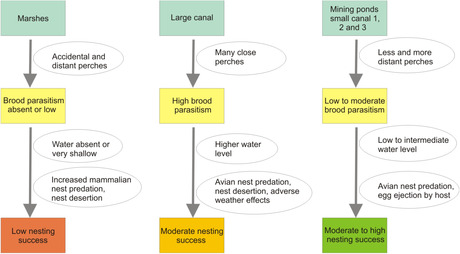

## 1 INTRODUCTION

One of the major ecological concerns in the last several decades is the human‐induced global loss of biodiversity by rapid extinction of species (Ceballos et al., 2015). By recognizing this global pattern, conservationists and scientists work on how to slow down and/or to counter the decline of threatened and endangered species (Bolam et al., 2021). At a regional scale, targeted restoration and management of habitats and landscapes may result in recovery of species richness and abundance increase that contribute positively to the global conservation status (Strassburg et al., 2020). Information about the spatial distribution of species and their abundance, and environmental variables influencing them are key elements in planning conservation actions (Rodrigues et al., 2007; Wiens et al., 2008). Appropriate management of habitats can create favorable conditions for targeted species ensuring the presence of both source and sink populations in meta‐populations (Dias, 1996). The identification and management of biodiversity hotspots help protecting vulnerable species with limited areal distributions (Myers et al., 2000). However, conservation science and practice should also focus on common, not only on rare or threatened, species, because common species can influence entire communities by their presence or abundance, indicating the quality of habitats, including biodiversity hotspots (Morelli et al., 2019).

Brood parasites are species that exploit the reproductive effort of their host species (Pollock et al., 2021) and because they depend and exert influence on several other species, their presence can indicate the quality of habitats. For example, the Common Cuckoo *Cuculus canorus* (hereafter Cuckoo) is considered an excellent bio‐indicator species as its presence predicts the hotspots of taxonomic and functional diversity of bird communities and the population changes in the Cuckoo mirrors climate suitability and overall trends of populations in bird communities (Morelli et al., 2017, 2019). However, we know little on the direct relationship between habitat quality/management and the presence or abundance of brood parasites. The effects of habitat management on the probability of brood parasitism and brood parasite offspring survival have been addressed only in a few North American studies that focused on how habitats should be managed in order to decrease the rate of parasitism of Neotropical song‐bird nests and prairie grassland bird by brown‐headed cowbirds *Molothrus ater* (Patten et al., 2006; Robinson et al., 1993). Although there are approximately 400 studies (Web of Science, Microsoft Academic) on brood parasitism by Cuckoos, none has addressed the effects of habitat management on Cuckoo brood parasitism, except for one small pilot study (Mérő & Žuljević, 2019). The latter study did not find a link between reed management and brood parasitism in a mining pond (Mérő & Žuljević, 2019), but we have no information from other habitat and management types. Understanding how habitat characteristics and management influence Cuckoo brood parasitism and reproductive success is important because recent studies detected a decline of Cuckoo populations in Europe (Denerley et al., 2019; Sparks et al., 2017), even though the species has a stable global population trend (BirdLife International, 2015). This decline, however, can be reverted by applying conservation measures in the breeding grounds to improve nesting habitats and food availability (Denerley et al., 2019; Hewson et al., 2016). Both nesting habitat quality and amount of food resources can be influenced by habitat management (e.g., Battisti et al., 2020; Lindstrom et al., 2020; Mérő et al., 2020). Habitat management can help in creating habitat fragments with vegetation patches of different ages (e.g., reed habitats), thus influencing the breeding density of potential Cuckoo hosts (Battisti et al., 2020; Mortelliti et al., 2012; Sozio et al., 2013).

The success of nest parasitism by the Cuckoos depends much on host–Cuckoo interactions, egg mimicry, and ability of egg recognition in the host (e.g., Marton et al., 2019; Procházka et al., 2010). However, we have little information on how habitat characteristics affect the breeding of Cuckoos. A significant factor is the availability and density of hosts (Procházka et al., 2010). However, host availability itself is not enough for successful parasitism. For example, ponds with high availability of reed edges and high host abundance attract a number of adult Cuckoos for breeding, but due to unfavorable habitat characteristics, host nests remain poorly parasitized, and those being parasitized have low nesting success because of egg ejection, predation pressure, and adverse weather circumstances, which can lead Cuckoos into an ecological trap (Mérő & Žuljević, 2019; Moskát et al., 2008). The availability and proximity of potential perches, such as solitary shrubs, trees, shrub, or tree‐rows, or electric wires near reedbeds are of crucial importance for the probability of nest parasitism (“perch proximity hypothesis”) (Antonov et al., 2007; Moskát & Honza, 2000; Øien et al., 1996). The perch proximity hypothesis proposes that brood parasites can better locate host nests when they have opportunities to observe the nesting host birds, for example, from branches of trees and shrubs, electric wires (Hauber & Russo, 2000). Closer perches provide better possibilities to detect host nests, which increases brood parasitism (Moskát et al., 2008). Besides perch availability, very few studies addressed the importance of host nest position and its surrounding vegetation (Moskát & Honza, 2000; Øien et al., 1996); thus, our knowledge on how these habitat characteristics (e.g., vegetation structure) affect brood parasitism and survival of parasitized nests is still poor. As opposed to the well‐concealed nests in dense vegetation, host nest positioned in sparse vegetation or higher in the reeds can increase the visibility and detectability of the nest by the brood parasite, thus increasing the risk for brood parasitism if perches are found in the surroundings (Mérő & Žuljević, 2019). Although habitat characteristics play an important role in the host nest detection of the brood parasite, a recent study found evidence that Cuckoos can follow their hosts alarm calls when the hosts defend their nests from predators, thereby they can obtain information on good nest parasitism opportunities (Marton et al., 2019). However, without perches in the vicinity of host nesting, the potential for brood parasitism decreases to a minimum, that is, while nests in habitats rich in perches are highly parasitized, nests in habitats without perches remain non‐parasitized (Moskát et al., 2008). Besides vegetation structure, several other habitat variables may influence Cuckoos' habitat selection and offspring success. Water availability, water depth, and water level fluctuation have been studied in the host, the Great Reed Warbler (Mérő et al., 2014, 2018, 2020). Earlier studies showed that water availability, the intermediate depth, and low water level fluctuation provide stable breeding circumstances, while the absence of water, or high water level, and/or large fluctuations decreased the survival of nests (Mérő et al., 2014, 2018). In addition, the number of parasitized nests was negatively related to the survival of Cuckoo nests in an earlier study of Mérő et al. (2013).

The aim of this study was to explore how habitat characteristics, management, and nesting variables influence the rate of brood parasitism and offspring survival in Cuckoos. We hypothesize that close perches, higher host density, less concealed nests, and nests located higher in reeds will increase the probability a nest being parasitized. Moreover, we expect that offspring survival in the Cuckoo will decrease with increasing chances of predation, such as when nests are located in sparse reeds, in reedbeds without water or very low or very high water level, or with highly varying fluctuations. Finally, we hypothesize that the number of parasitized host nests and nesting success of Cuckoo are independent from each other because the factors influencing nest selection and chick survival will be different. We studied Cuckoo brood parasitism in nests of the Great Reed Warbler *Acrocephalus arundinaceus*, a common host of the Cuckoo in the lowlands of Central and Eastern Europe. We specifically assess how vegetation management can influence the rate of brood parasitism in different reed habitat types (mining ponds, marshes, large canals, and different classes of small canals) distributed in an intensive agricultural landscape. We also evaluate how habitat management, vegetation structure, and water variables influence the survival of Cuckoo offspring in parasitized host nests. We use the results to develop conservation recommendations on how to improve the survival of Cuckoo parasitized clutches, in order to increase the abundance/density of regionally declining Cuckoo populations.

## 2 MATERIAL AND METHODS

### 2.1 Study area

**FIGURE 1:**
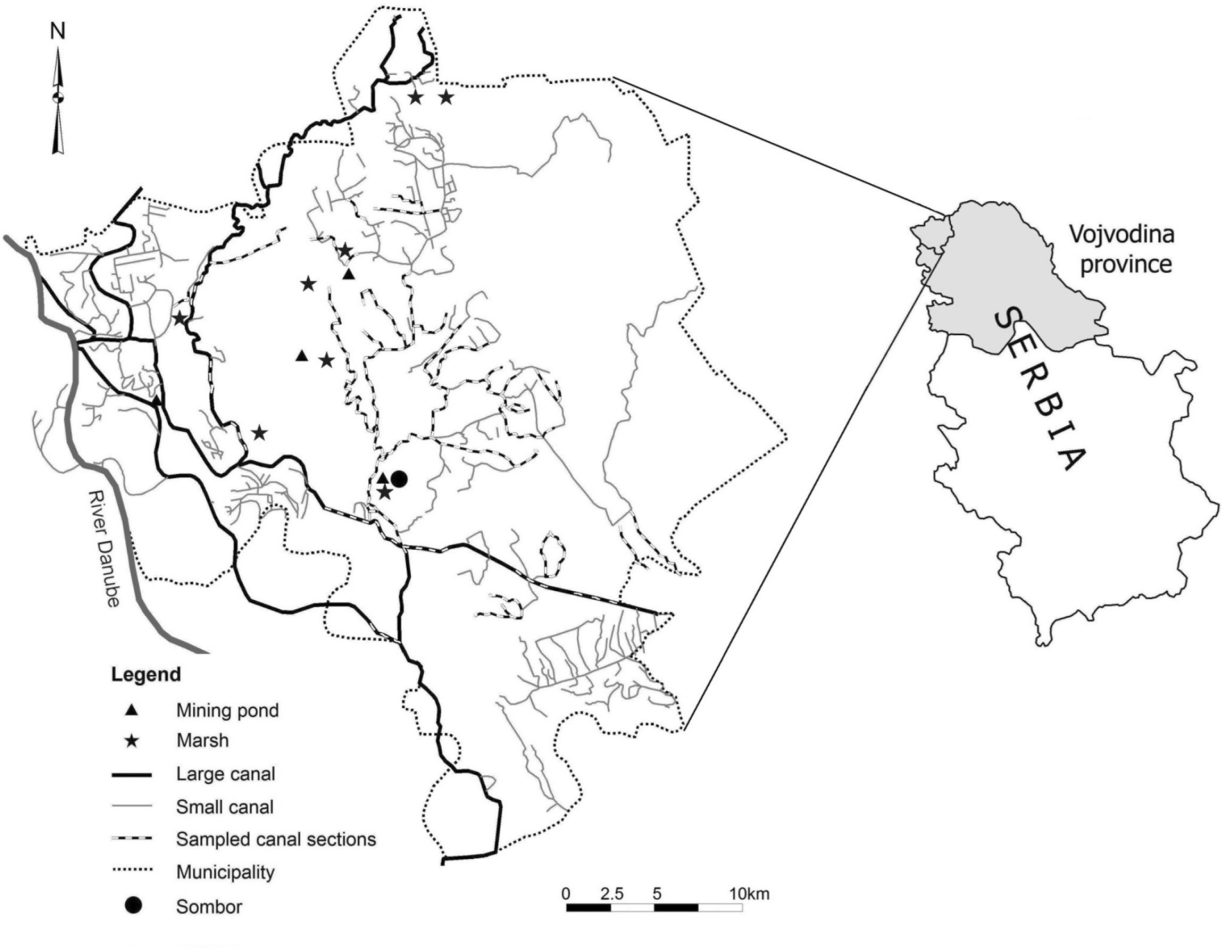
The sampled large canal and small canal sections (stripes), marshes (stars), and mining ponds (triangles) in the county of Sombor, NW Serbia. The figure shows all sections of canals in which fieldwork was performed in any of the 13 study years.

We conducted this study in 45 different study sites (hereafter reedbeds) that represented six types of reed habitat (hereafter reed habitats) distributed in an intensive agricultural region in the county of Sombor (1178 km^2^, N 45°, E 19°, NW Serbia). Reed habitats were mining ponds, marshes, large canals, and three size classes of small canals (Figure 1; Table 1).

**TABLE 1:** Mean ± SD values of reed management variables, perch availability, nest variables, water variables, and reed density for the six studied reed habitats based on the total number of Great Reed Warbler nests (including Cuckoo parasitized nests), and only on Cuckoo parasitized nests, both combined from 13 years.

Reed habitat (*n*)	Mean values (mean ± SD): Great Reed Warbler nests					
	Proportion of managed reed (%)	Julian date of reed management (days)	Perch availability^1^	Nest density (ha^−1^)	Nest height (cm)	Mixed reed density (m^−2^)
Mining ponds (4)	27.9 ± 37.8	132.1 ± 156.7	2.0 ± 1.1	11.9 ± 6.8	81.2 ± 26.9	227.3 ± 31.5
Marshes (8)	12.5 ± 35.3	37.5 ± 106.1	0.0 ± 0.0	4.9 ± 5.0	91.9 ± 24.8	252.7 ± 80.3
Large canals (8)	13.5 ± 13.6	197.3 ± 139.3	3.9 ± 0.3	5.9 ± 2.9	108.1 ± 8.5	196.8 ± 44.7
Small canals 1 (10)	55.2 ± 46.6	125.6 ± 116.8	2.3 ± 1.0	17.2 ± 14.2	98.8 ± 21.5	193.2 ± 43.4
Small canals 2 (10)	48.0 ± 48.0	151.0 ± 155.6	1.6 ± 0.8	8.6 ± 7.2	96.5 ± 10.0	192.2 ± 31.3
Small canals 3 (5)	0.5 ± 0.2	132.0 ± 102.0	1.5 ± 0.7	9.6 ± 4.9	89.3 ± 4.1	168.0 ± 1.4

	Mean values (mean ± SD): Cuckoo parasitized nests					
	Proportion of managed reed (%)	Julian date of reed management (days)	Nest height (cm)	Water depth (cm)	Water level fluctuation (cm)	Mixed reed density (m^−2^)
Mining ponds (3)	19.9 ± 32.8	103.9 ± 149.5	85.6 ± 26.9	44.7 ± 41.5	−4.5 ± 14.5	242.2 ± 100.2
Marshes (3)	10.0 ± 20.0	75.0 ± 150.0	82.5 ± 27.8	16.23 ± 14.1	−9.0 ± 1.7	286.6 ± 139.9
Large canals (6)	10.7 ± 11.5	180.3 ± 134.1	105.2 ± 31.6	60.5 ± 32.3	−1.4 ± 5.6	186.2 ± 113.8
Small canals 1 (7)	58.5 ± 41.7	133.1 ± 97.5	99.1 ± 30.6	27.4 ± 26.6	−12.0 ± 10.6	180.3 ± 81.5
Small canals 2 (5)	58.8 ± 47.9	130.0 ± 121.1	102.8 ± 31.5	14.3 ± 19.8	−10.5 ± 11.7	161.5 ± 106.5
Small canals 3 (1)	0.0 ± 0.0	0.0 ± 0.0	91.0 ± 31.1	13.2 ± 8.8	−1.7 ± 2.9	177.5 ± 40.0

The mining ponds (*n* = 4 reedbeds) were established until the 1970 s by clay excavation by the local brickyards. The surface of these ponds is relatively small, varying between 0.7 and 2.0 ha, and is covered with patchy, fragmented, or closed, mainly homogeneous reedbeds. The amount of water and its variation depends strongly on the amount of precipitation in the winter and spring, and on the variation of the groundwater table, which usually decreases throughout the summer and early autumn droughts and agricultural irrigation. The reedbeds are often burnt in February and March by the locals, resulting in patchy and mosaic pattern of reed burn.

Successional marshes (*n* = 8) are remnants of winding and sluggish lowland rivers whose water supply ceased after river regulations in the 19th century. The size of marshes ranges between 2 and 13.5 ha. They are usually covered by closed and often homogeneous reedbeds, except for two marshes on salty soil, which are covered by patchy reedbeds. The water level is influenced by the amount of precipitation. In most years, water generally disappears in June due to high temperatures and extensive evaporation of the vegetation. Marshes are practically not managed.

The large canals (*n* = 8; total length of sampled sections 14.3 km; mean length 1.8 ± 1.56 SD km) function as the leading water bodies of the canal hydro‐system in the region. Their width varies between 15 and 35 m, and their banks are covered with 2‐ to 6‐m wide reed strips, that are often interspersed with shrubs or shrub rows of mainly *Salix*, *Typha*, and/or *Carex* species. Water level displays only small to moderate fluctuations, because of active regulation through a sluice system. Occasionally some sections of reed stands are burnt by locals or mown by the water management company to reduce the accumulation of vegetation litter on banks.

Small canals were classified into three size categories by the water management company, according to their width, drainage capacity, and area of the water catchment basin. Small canals 1 (*n* = 10; total length of surveyed sections: 34.2 km; mean length 3.8 ± 2.59 km) are usually deep, with a width of approximately 6 m. Until 2015, the reedbed patches on both sides of the banks were mown in late summer and autumn. Later reed was managed by vegetation clearing during autumn and winter (Buisson et al., 2008). Management was applied in the autumn or winter once a year or in every other year, depending on the needs of water management, or when adverse weather caused difficulties in water drainage. The purpose of linear vegetation management was to facilitate the drainage and water supply function of the small canals. The width of small canals 2 (*n* = 10; 41.2 km; 3.2 ± 1.53 km) varies from 2 to 4 m, and patchy reed stands stretch over the canal channel. Small canals 2 are characterized by intermediate water depth, and intermediate decrease of the water level during the breeding season (Table 1). The irregularly managed reed was mainly mown, either on one or both sides. Finally, small canals 3 (*n* = 5; 10.5 km; 2.1 ± 0.88 km) are shallow, vary in width from 1 to 3 m, and contain patchy reedbeds, which are rarely managed. These shallow canals usually dry out already by late May or early June. For more information about the study region and reed habitats, see Mérő et al. (2016, 2018, 2020).

Cuckoo perches are often solitary shrubs, trees, or electric wires, or high banks located in the vicinity of the reedbeds (Moskát & Honza, 2000). In the surrounding of the reedbeds of mining ponds, the nearest potential perches for Cuckoos are usually solitary trees and shrubs. In case of the marshes, the relatively far located (>25 m) trees, tree rows, shrubs, or electric wires may serve as potential perches for the Cuckoo. The electric wires and the forest belts on both sides of the large canals provide excellent perches close to the reedbed. Potential perches for Cuckoos were similar to those near small canals 1. In case of small canals 1 and 2, Cuckoo perches such as solitary shrubs, trees, and electric wires are relatively close to the reedbeds. Potential perches such as solitary shrubs or solitary trees are rare and far from the reedbeds of the small canals 3.

### 2.2 Sampling methods

Fieldwork was conducted from April to August in 13 years (2008–2020), with a similar effort and intensity during the entire Great Reed Warbler and Cuckoo nesting seasons in every study year. Data on perch availability, nest height, water variables, and reed density were collected during the nest searches and nest checks in each reedbed. In mining ponds and smaller marshes, the entire reedbed was searched for nests, while in larger marshes, nests were searched in randomly selected units of the reedbed. In canals, we searched for nests systematically by walking on both banks or from a boat (Sevylor Colorado canoe) from the water in randomly selected sections. Nests were monitored once every 5 days to assess nest fate and/or the number of both host and parasitic chicks surviving to fledgling. During the nest checks, we recorded the number of host and parasitic eggs, the number host and parasitic eggs hatched, the number of host and parasitic chicks, and the number of successfully fledged host and parasitic chicks. In case of egg and/or nestling loss, we recorded the cause of failure (predation, desertion of nest, ejection of the parasitic egg by host, human activity, or adverse weather; Table 2). In order to minimize the chances of discovery of the host nests by Cuckoos and predators, we repositioned reed stalks after every nest check to restore their density and position to the status in which we had found them. A nest was considered to be parasitized when nests contained Cuckoo egg(s) and/or chick(s). Nest desertion was inferred when we found cold eggs with or without damage and/or dead chicks in the nest on at least two nest check occasions. Egg ejection was defined when the Cuckoo egg was removed (ejected) from the nest by the host, regardless of the later fate of the nest (fledging, predation, or desertion). Egg or nestling eviction was defined as the removal of host and parasitic eggs and/or nestlings by a Cuckoo nestling. A successfully fledged Cuckoo chick was recorded if the young was seen sitting on edge of the nest, or on reed stems in the vicinity of the nest, or when large amount of feather dust and excrement was found in the nest or on reed stalks around the nest. We developed the sampling methods according to the needs of the current study, based on Øien et al. (1998), Moskát and Honza (2000), Mérő et al. (2014, 2015, 2020).

**TABLE 2:** The number of Great Reed Warbler nests and number of Cuckoo‐parasitized nests, and fledgling success (Mayfield nesting success), and proportion of nests lost by predation, desertion, egg ejection, and other factors in each reed habitat.

Reed habitat	Number of Great Reed Warbler nests	Number of Cuckoo parasitized nests (%)	Success of Cuckoo nests^a^	Proportion of nests lost by			
				Predation	Desertion	Egg ejection	Other^b^
Mining ponds	328	33 (10.1)	0.377	0.45	0.03	0.15	0.00
Marshes	65	4 (6.1)	0.239	0.50	0.25	0.00	0.00
Large canals	382	153 (40.0)	0.411	0.24	0.18	0.08	0.06
Small canals 1	417	61 (14.6)	0.439	0.21	0.08	0.13	0.15
Small canals 2	286	49 (17.1)	0.550	0.14	0.14	0.06	0.10
Small canals 3	56	3 (5.4)	1.000	0.00	0.00	0.00	0.00
Total	1534	303 (19.7)	0.440	0.24	0.14	0.10	0.08

^a^ estimated according to the improved Mayfield (1975) method: Johnson (1979) and Hensler and Nichols (1981).

^b^ reed mowing; reed burning; tree cutting; Cuckoo young fell out of nest and drowned; unknown.

We characterized reed habitat quality by two variables for habitat management (proportion of managed reed, and Julian date of reed management), by four variables for reedbed quality (perch availability, mixed reed density, water depth, and fluctuation of water level), and two variables for nesting (nesting density and nest height; Table 1). Each of these eight variables was assessed in each reedbed in each study year. We measured the managed and non‐managed parts of the entire area of the reedbeds by walking in managed and non‐managed patches with a GPS device. We quantified reed management as the proportion of the managed area per the entire reed bed. To assess the timing of reed management, we used the Julian date as the number of days passed since May 1 every year. In cases when management was applied after the nesting season and/or during autumn (hereafter “pre‐winter management”), its effects were considered to affect the breeding in the subsequent year. The effects of late management, applied during late winter and spring until the end of April (hereafter post‐winter management), was considered to affect the upcoming breeding season. Pre‐winter management was usually done by mowing or complete vegetation removal (including the reed stems), while post‐winter management was usually done by vegetation removal by the water management authorities, and reed burning by the local people. We recorded all potential perches (shrubs, trees, electric wires) used by Cuckoos and located within 10 m from the reedbed using a GPS. According to the perch availability, reedbeds were classified into five categories: 0—no perches available; 1—one or two solitary shrubs or trees available; 2—several (three to eight) solitary shrubs or trees available; 3—shrubs and trees appear frequently; 4—continuous presence of shrub and trees in form of rows and/or forest belts, and electric wires. For all reed habitats, we decided to calculate the density of nests for surface area including mining ponds, marshes, and all types of canals. This was reasonable because in marshes, the Great Reed Warbler nests were often distant from edges, or in some cases, the marsh did not contain reed edges adjacent to water. Therefore, nest density calculated per reed edge length was not possible. In the calculation, we included the studied area of the reedbed surface and water surface. The host nest density for each reedbed was calculated for 1 ha surface area, with the consideration of all Great Reed Warbler nests (including parasitized nests) found in the reedbed. Nest height was measured as the distance between the ground or water surface and the bottom of the nest cup. We recorded reed density by counting both old and new reed stalks in a circle of 50 cm in diameter placed 1 m from the nest in a random direction, and we then extrapolated this to 1 m^2^. We estimated the density of mixed (old and new) reed, typically used by Great Reed Warbler for nesting (Table 1). We used a measuring stick (accuracy: 5 cm) to determine water depth. The fluctuation of water level was calculated as the difference between the maximum and minimum measured water depths during the breeding season.

### 2.3 Statistical analysis

Parasitism rate, that is, the proportion of Cuckoo parasitized nests was calculated as the number of parasitized nests divided by the total number of host nests found in a reedbed in a breeding season (Moskát & Honza, 2002). The nesting success of the Cuckoo per nest, that is, the probability that an egg produces a fledgling, was calculated by the improved Mayfield (1975) method (Hensler & Nichols, 1981; Johnson, 1979). To explore the differences in the daily survival rate (DSR) of the nest in the egg and in the nestling stage, we applied the J‐test (Hensler & Nichols, 1981; Johnson, 1979). Similarly, to compare parasitized nests in the early versus the late parts of the nesting season, we divided nests as early and late at the median date (Julian days) between the dates of finding the first and the last nests in the season. The early period lasted from the second half of May to the first half of June, whereas the late period lasted from the second half of June and first half of July. We applied the J‐test to compare survival rates between the early and late nesting period (Hensler & Nichols, 1981; Johnson, 1979). We used linear regression to test the relationship between parasitism rate and the number of successful Cuckoo nests, defined as a nest that produced at least one Cuckoo fledgling.

In order to test the effects of reed habitat type, reed habitat quality (perch availability, reed density, water depth, water level fluctuation), reed management (proportion of managed reed, Julian date of management), and nesting variables (host nest density, nest height) on parasitism rate and survival of parasitized nests (hereafter nesting success); we constructed two linear generalized mixed‐effects models (GLMM1 and GLMM2). In GLMM1, we used data from all host nests found, while in GLMM2, we only used data from parasitized nests. Perch availability, host nest density, and reed density were used only in GLMM1 as these variables were not expected to influence nesting success after the nest was parasitized, whereas water level decrease was used only in GLMM2 as seasonal water level decrease was expected to influence nesting success but not the parasitism of nests, most of which occurs earlier in the season. In GLMM1, we modeled the relationship between parasitism rate (response variable) and reed habitat type, perch availability, reed density, water depth, proportion of reed management, Julian date of management, host nest density, and nest height (fixed effects), with “year” and “reedbed” as random factors to control for annual, weather‐related variation at each site. In GLMM2, we tested the relationship between nesting success (response variable) and reed habitat type, water depth, water level decrease, nest height, proportion of reed management, and Julian date of management (fixed effects), with “year” nested within “reedbed” (random factor). Before running models, we standardized each continuous predictor variable to range between 0 and 1. Because parasitism rate was a proportional variable based on counts, we built GLMM1 by using the “glmmTMB” function in R with binomial distribution as recommended in Douma and Weedon (2019). Similarly, because Cuckoo chick survival was a proportional variable based on a continuous estimator variable, we used the “glmmTMB” function with beta distribution in GLMM2, as recommended in Douma and Weedon (2019). Before GLMM2, we applied the following data transformation suggested for data containing zeroes and ones (Douma & Weedon, 2019):

$$p^{*}=\frac{p\left(n-1\right)+1/C}{n}$$

where *p* is the DSR of Cuckoo chick(s), *n* is the total number of observations, and *C* is the number of categories (2 in our case).

We first built full models with main effects and relevant interactions for both GLMM1 and GLMM2 and applied a backward stepwise removal of nonsignificant (*p* > .1) interactions and nonsignificant (*p* > .05) main effects to obtain minimum adequate models, which then we used to estimate coefficients. The relevant two‐way interactions tested were between reed management variables on one side and habitat quality and nesting variables on the other side (*n* = 10 interaction terms in GLMM1, *n* = 6 terms in GLMM2). To check the differences in parasitism rate among the reed habitat types, we calculated coefficient estimates for each reed habitat relative to large canals, where we expected the largest parasitism rate based on previous findings (Mérő et al., 2013; Moskát & Honza, 2002).

The normality of the variables was checked with Shapiro–Wilk tests, and the homogeneity of variances with Bartlett tests. We fitted linear mixed models (LMM) with Gaussian error distribution and identity link by the “glmmTMB” function of the “glmmTMB” R package (version 3.6.3., R Core Team 2020). This function uses an iterative process based on maximum likelihood, allows unequal variances, and is robust to unbalanced designs (Bates et al., 2015).

## 3 RESULTS

We found and monitored 1534 Great Reed Warbler nests, of which 303 (19.7%) were parasitized by Cuckoos (Table 2). In 273 parasitized nests (91%), we found one parasitic egg or nestling. We found two parasitic eggs in 24 nests, and three parasitic eggs in six nests. Of these multiply parasitized nests, 24 (80%) were in the large canals. In 24 multiple parasitized nests, we recorded predation or desertion, while in four nests, we observed the eviction of four Cuckoo eggs by the host, and in two cases, the eviction of two Cuckoo chicks by their siblings.

**FIGURE 2:**
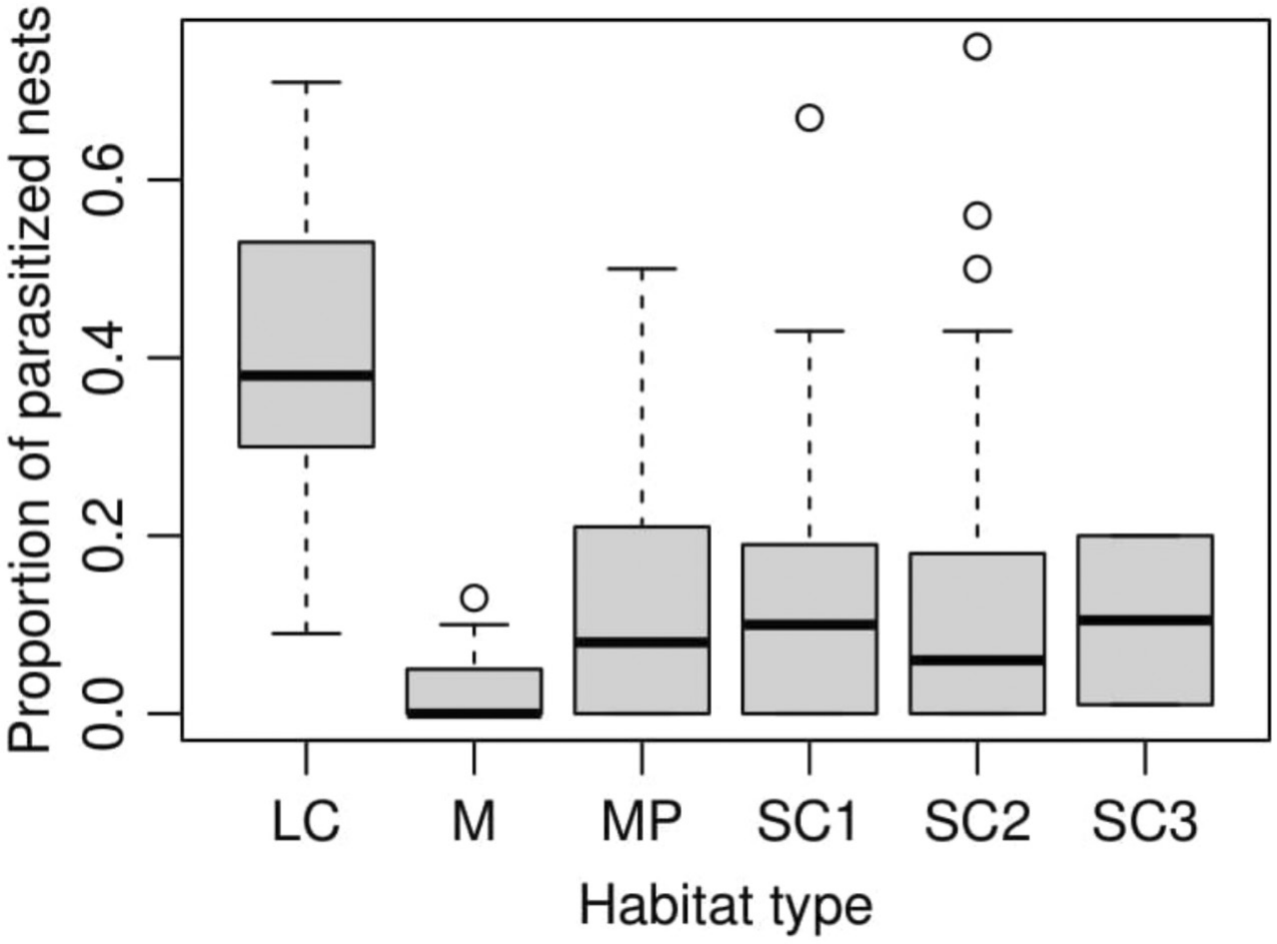
The proportion of Great Reed Warbler nests parasitized by Common Cuckoos as a function of habitat type (LC—large canals, M—marshes, MP—mining ponds, SC—small canals of 3 size categories from larger to smaller).

**FIGURE 3:**
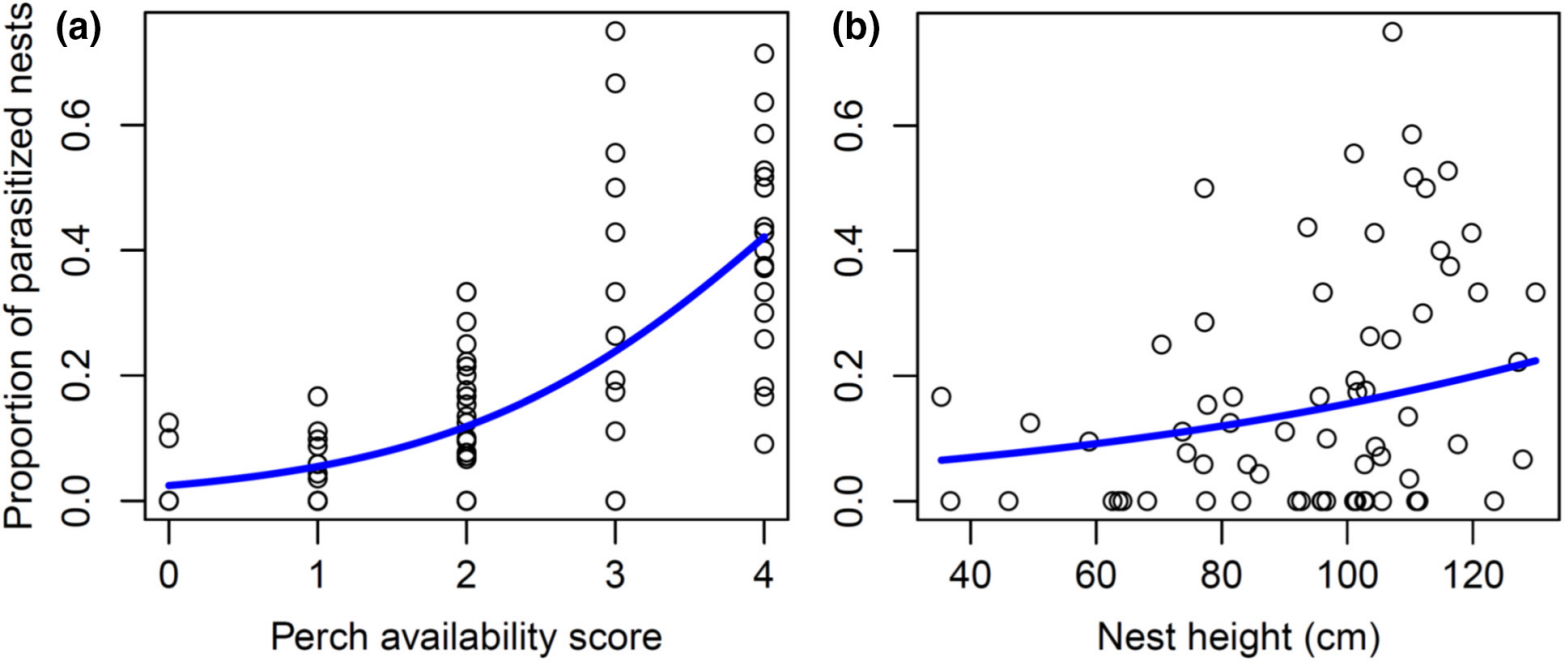
The proportion of Great Reed Warbler nests parasitized by Common Cuckoos as a function of the availability of Cuckoo perches (a) and the height of the nests (b).

LMM1 showed that parasitism rate differed among reed habitats (Figure 2; Table 3), mostly because it was very high (40%) on large canals and much lower (5%–17%) in other habitat types (Table 2; χ^2^ = 141.80, df = 5, *p* < .0001). The proportion of Cuckoo‐parasitized nests was also positively related to the availability of perches and marginally nonsignificantly to nest height (Figure 3a,b; Table 3). The random variation in intercepts across the locations (2.017 × 10^−3^) explained several orders of magnitude more variation in parasitism rate than the random variation in intercept across years (3.164 × 10^−10^). Finally, none of the interactions between main effects was significant, and brood parasitism rate was not affected by Julian date of management, host nest density, or reed density.

**TABLE 3:** Coefficient estimates from linear mixed‐effects minimum adequate models testing the effects of reed habitat characteristics and reed management (fixed effects) and year and study site (random factors) on the proportion of Great Reed Warbler nests parasitized by Cuckoos (GLMM1) and on Mayfield nesting success of Common Cuckoos in parasitized nests (GLMM2).

Model	Variable	Estimate ± S.E.	Df	Test statistic	*p*
GLMM1	(Intercept)	−5.37 ± 0.744	1	z = −7.222	<.0001
	Habitat type		4	χ^2^ = 11.999	.017
	Marsh—Large canal	1.86 ± 0.793	17	z = 2.345	.019
	Mining pond—Large canal	0.43 ± 0.344	17	z = 1.261	.207
	Small canal 1—Large canal	0.16 ± 0.313	17	z = 0.496	.620
	Small canal 2—Large canal	0.89 ± 0.322	17	z = 2.764	.006
	Perch availability	0.85 ± 0.128	42	χ^2^ = 44.012	<.0001
	Nest height	1.32 ± 0.764	42	χ^2^ = 2.992	.084
GLMM2	(Intercept)	0.52 ± 0.153	1	z = 3.380	.0007
	Water depth	−0.91 ± 0.369	207	χ^2^ = 6.043	.014

Cuckoo young successfully fledged from 135 nests (44.6%, *n* = 303). The survival of Cuckoo eggs and nestlings was limited by nest predation, nest desertion, egg ejection by the host, and other factors (adverse weather, human activities such as reed mowing or burning, tree cutting, and recreational angling) (Table 2). The number of parasitized nests was not related to the number of successful Cuckoo nests (slope = −0.484 ± 0.998, *F* = 0.234, numerator df = 1, denominator df = 4, *p* = .654). The daily survival rate was marginally nonsignificantly higher in the nestling stage than in the egg stage (DSR_eggs_ = 0.969, DSR_nestlings_ = 0.978; z = 1.955, *p* = .050). Parasitized nests found in the later period had significantly higher survival rate (DSR = 0.494, *n* = 129 nests) than those in the earlier period (DSR = 0.383, *n* = 174 nests; *j*‐test, *z* = 2.086, *p* = .037). After egg ejection by the host, at least one Great Reed Warbler young fledged successfully in 15 nests (52%, *n* = 29 nests), while 10 nests (34%, *n* = 29) were depredated and four nests (14%, *n* = 29) were deserted or lost due to human activity or adverse weather.

**FIGURE 4:**
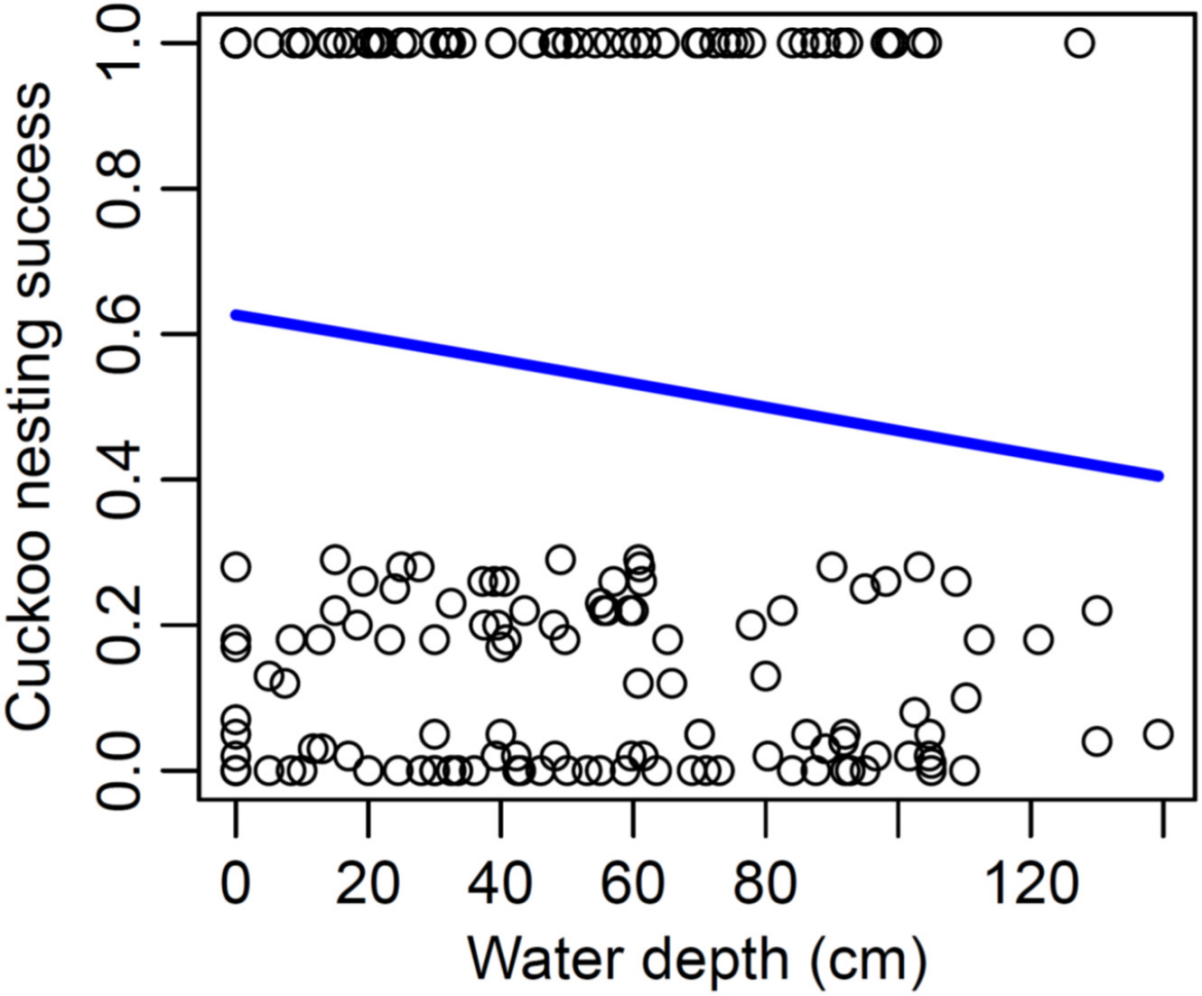
Cuckoo nesting success (Mayfield‐estimated chick survival) as a function of water depth.

In LMM2, we found that water depth negatively affected nesting success (Figure 4; Table 3).

The random variation in intercepts across the years (4.433 × 10^−9^) was two orders of magnitude higher than the random variation in intercepts across the locations (6.173 × 10^−11^). None of the other predictor variables or the interactions between them had a significant effects on nesting success (Table 3).

## 4 DISCUSSION

Our results show that habitat‐dependent nest detectability by Cuckoos was primarily important in explaining brood parasitism rates. Perch availability positively influenced nest parasitism, indicating that nests built in reeds in areas with more perches are more exposed and more likely to be parasitized by Cuckoos, confirming our expectations based on the perch availability hypothesis. Parasitism rate was also weakly influenced by nest height. This is not surprising because nests built higher are even easier to detect in areas with many available perches. The number of successful nests was not related to the rate of parasitized nests, confirming our expectations. Nesting success was negatively affected by water depth, indicating that eggs and nestlings were more vulnerable in reed habitats with deeper water, also confirming our expectations.

Although a pilot study on a mining pond (Mérő & Žuljević, 2019) did not find a relationship between perch distance and probability of parasitism, our results from several habitat types in general supported the “perch proximity” hypothesis (Antonov et al., 2007). Earlier studies suggested that the shape of the reed bed, position of the host nests (Mérő & Žuljević, 2019), and perch distance from nests play an important role in parasitism rate among different reed habitats (Moskát et al., 2008). The differences found in parasitism rate among reed habitat types might also arise because of similar factors. While in linear reed habitats such as canals, perches play an important role (Moskát et al., 2008, this study), in reed habitats with wide reed vegetation such as in marshes, the distant perches have rather negligible effects (Moskát et al., 2008). Similar patterns were also found in other *Acrocephalus* warblers. For example, in the Marsh Warbler *A. palustris*, the combination of perch height, perch proximity, and nest location influenced parasitism rate (Antonov et al., 2007). In our study, nest height was exerting a weakly positive effect on parasitism rate, suggesting that nests placed higher among the reeds are easier to detect by Cuckoos. In the North American brown‐headed cowbird, nest parasitism rate also increased with the height of the nests (Larison et al., 1998). There can be spatial and temporal variation in nest height, which can have negative effects on brood parasitism. In years with less water availability, the hosts often build nests farther from the banks (and perches), and even though those nests might be built higher, they can be more difficult for Cuckoos to find (Mérő & Žuljević, 2019). It is thus plausible that nest height is related to water availability such that in deep water, nests are built closer to water level, while in shallow water or dry reed beds, nests are usually built higher.

Overall, our results showed that different reed habitats can have considerable differences in parasitism rate depending on the availability of perches and nest position (Table 2). We note that besides the standard perches such as shrubs, trees, or electric wires, we also observed adult Cuckoos using newly mown higher banks of small canals I as potential perches. Our observations supported the view the higher the availability of perches, the higher the number of female Cuckoos present (Moskát et al., 2019). Cuckoos prefer habitats with ample opportunities for nest parasitism and which offer good conditions for offspring survival (Vogl et al., 2002). Our finding of multiply parasitized nests (eggs laid by two or more females in the same host nest) in reed habitats with continuous perch availability (Marton, 2021) supported this view. In general, our results show that perch availability and nest height in reed beds are crucial factors in determining the probability of brood parasitism.

Similar to the nesting success of host nests (Mérő et al., 2014; Mérő & Žuljević, 2016), the Cuckoo nesting success was also moderate in deeper water, and lower in reedbeds with shallow or no water (marshes). In the case of Cuckoo, this is likely explained either by higher detectability of Cuckoo‐parasitized nests by predators or by lower suitability of the reed stems to hold the nest with a heavy Cuckoo chick. First, reedbeds with higher water level, the sparse reed probably offers better chances for snake or avian predators to detect the nests. The intensive loud call of the Cuckoo chick may also attract predators more intensively than the silent begging of the host chicks (McDonald et al., 2009). In marshes with shallow or no water, Cuckoo chick predation was more frequent because besides bird predators, mammal predators could also approach the nests. Evidence for such increased predation of begging Cuckoo chicks was found in Reed Warbler *Acrocephalus scirpaceus* but not in Great Reed Warbler (Jelínek et al., 2016). Nevertheless, in areas of high predation (mainly marshes and large canals), we regularly observed bird predators such as the Night Heron *Nycticorax nycticorax*, Purple Heron *Ardea purpurea*, Little Bittern *Ixobrychus minutus*, and the Hooded Crow *Corvus cornix*, and mammalian predators such as the Red Fox *Vulpes vulpes* and Least Weasel *Mustela nivalis* (T. O. Mérő, A. ŽDuljević, D. Malbaša, & S. Lengyel, in preparation). Although mammalian predation as a risk on nest survival can be almost excluded due to deep water level on large canals, the weather conditions and the host defense mechanisms against parasitism in highly parasitized populations reduce survival probability in the Cuckoo (Moskát et al., 2008).

Second, the growth of the reed in deep water in the spring and summer period is reduced, resulting often weaker, shorter, and sparser reed stems (Graveland, 1998), that may be less suitable for holding the nest with a Cuckoo chick that eventually grows several times larger than the host adults themselves. Such reed stems may be more susceptible to adverse weather than mixed reed stems in reedbeds with low to intermediate water depth, such as the mining ponds, and small canals 2 and 3. In later stages of Cuckoo chick development, nests carrying the heavy Cuckoo chick on sparse, thin reed stems may fall into the water or on the ground. This happens usually in the first half of the breeding season, when fresh reed stems are not hard enough and cannot resist strong winds or heavy rain (Mérő et al., 2013) or just simply collapse under the weight.

### 4.1 Conservation implications

**FIGURE 5:**
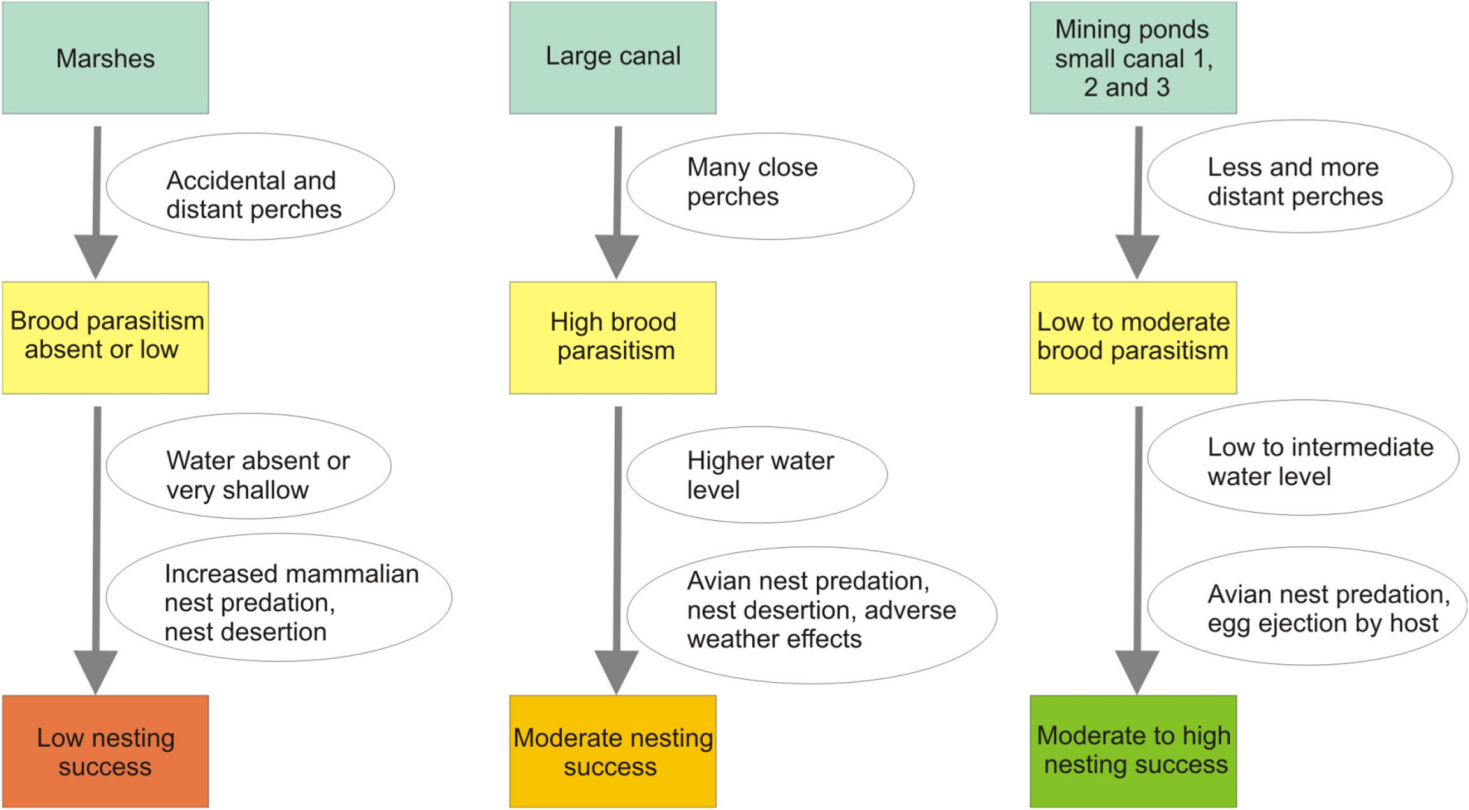
The conceptual framework displaying the cause–effect relationships among the factors and targets. Squares display the different stages in the process and the end stations, and circles display the factors influencing the stages and end stations.

Although the rate of brood parasitism was the highest on large canals, in light of Cuckoo nest selection and nesting success, reed habitats with many potential perches and low to intermediate water level (such as in mining pond, and small canals 2 and 3) appear to provide the best chances for successful breeding for Cuckoos (Figure 5). While parasitism rate can be lower in such habitats, the survival of both the parasitic and host chicks will be higher, because of the availability of strong reed that can hold nests in adverse weather conditions. The regulation of water level (depth) should be carefully applied in the different reed habitats, in order to maximize Cuckoo nesting success. Inappropriate water‐level regulation can result in the growth of weak, sparse, and thin reed stems, that is, directly influencing reed quality (Graveland, 1998). Good quality reedbeds, containing stronger old and new reed stems, can maximize the number of quality hosts (Mérő et al., 2016, 2018, 2020; Mortelliti et al., 2012), and increase Cuckoo nesting success. In marshes and mining ponds, we suggest the maintenance of intermediate water depth if possible (irrigation or supplying of water in coordination with water management companies), because the early nests of the host are usually placed in the mixed reed stands in intermediate water levels (Figure 4), providing stability and better concealment (e.g., Brambilla et al., 2020). In canals, we suggest to provide low to intermediate water depth at reed edges adjacent to water, ensuring the growth of appropriate quality reed for breeding. The supplying of the reedbeds with water should be applied at the end of winter, when reed shoots start their intensive growth. In general, high water level and absence of water in the reedbeds should be avoided. With such water level maintenance, we can increase the host occupancy of the reed habitats and also provide good conditions for chick rearing for both the host and the parasitic species. By maintaining Cuckoo populations, we can exert positive effects on animal species richness and abundance, which can contribute to the establishment of biodiversity hotspot patches in the landscapes (Morelli et al., 2017).

## AUTHOR CONTRIBUTIONS

**Thomas Oliver Mérő:** Conceptualization (lead); data curation (equal); formal analysis (equal); funding acquisition (supporting); investigation (lead); writing – original draft (lead); writing – review and editing (equal). **Antun Zuljevic:** Conceptualization (supporting); investigation (supporting); writing – review and editing (supporting). **Szabolcs Lengyel:** Conceptualization (supporting); data curation (equal); formal analysis (equal); funding acquisition (lead); investigation (supporting); project administration (lead); writing – original draft (supporting); writing – review and editing (equal).

## CONFLICT OF INTEREST

The authors declare no competing interests.

## Data Availability

The data that support the findings of this study are openly available in Dryad at: https://doi.org/10.5061/dryad.sbcc2fr90.
